# Mineral Analysis of Pine Nuts (*Pinus* spp.) Grown in New Zealand

**DOI:** 10.3390/foods2020143

**Published:** 2013-04-03

**Authors:** Leo P. Vanhanen, Geoffrey P. Savage

**Affiliations:** Food Group, Department of Wine, Food and Molecular Biosciences, Lincoln University, Lincoln 7647, Canterbury, New Zealand; E-Mail: savage@lincoln.ac.nz

**Keywords:** pine nuts, *Pinus* spp., mineral content, diet

## Abstract

Mineral analysis of seven *Pinus* species grown in different regions of New Zealand; Armand pine (*Pinus armandii* Franch), Swiss stone pine (*Pinus cembra* L.), Mexican pinyon (*Pinus cembroides* Zucc. var. bicolor Little), Coulter pine (*Pinus coulteri* D. Don), Johann’s pine (*Pinus johannis* M.F. Robert), Italian stone pine (*Pinus pinea* L.) and Torrey pine (*Pinus torreyana* Parry ex Carrière), was carried out using an inductively coupled plasma optical emission spectrophotometer (ICP-OES) analysis. Fourteen different minerals (Al, B, Ca, Cr, Cu, Fe, K, Mg, Mn, Na, Ni, P, S and Zn) were identified in all seven varieties, except that no Al or Na was found in *Pinus coulteri* D. Don. New Zealand grown pine nuts are a good source of Cu, Mg, Mn, P and Zn, meeting or exceeding the recommended RDI for these minerals (based on an intake of 50 g nuts/day) while they supplied between 39%–89% of the New Zealand RDI for Fe. Compared to other commonly eaten tree-nuts New Zealand grown pine nuts are an excellent source of essential minerals.

## 1. Introduction

Pine nuts (*Pinus pinea* L.) have been used in European cooking for a long time as they are highly valued as ingredients in pesto, sauces and as a garnish in desserts. The main species consumed in Europe are *Pinus pinea*, *P. koraiensis*, *P. sibirica* and *P. gerardiana*. As local production in Europe is costly and not sufficient to meet current demand, most commercial products are imported from China, Korea and Pakistan where the climate is suitable for efficient production. It is estimated that 36,080 tonnes of pine nut kernels were produced globally in 2011. China and the Russian Federation are the largest producers contributing 55% and 14%, respectively, to the world production. In 2010, New Zealand imported 22 tonnes of pine nut kernels for domestic consumption [1].

Pine nuts are relatively new crops in New Zealand and is a growing sector of the tree nut industry particularly in areas of the country with a favourable macroclimate and soil type. The mineral profile of *P. pinea* nuts grown in New Zealand or Australia has not been reported previously. Internationally there are reports on the mineral profile of *P. pinea* nuts from Portugal [2], Spain [3] and Turkey [4]. Evaristo *et al.* [2] observed significant differences in the mineral profile and other chemical components of pine nuts grown in different regions which suggest that environment and soil types have an important influence. When the mineral data for nuts grown in Portugal were compared to the data from Turkey, Evaristo *et al.* [2] observed that there was a substantial variation in the composition of all minerals measured. The authors also proposed that this variation may be useful to discriminate between Mediterranean populations of *P. pinea*.

In this study a complete mineral analysis of seven different species of pine nuts currently being grown in New Zealand was carried out and these results were compared to a sample of imported pine nuts.

## 2. Experimental Section

### 2.1. Materials

Seven *Pinus* species, Armand pine (*Pinus armandii* Franch), Swiss stone pine (*Pinus cembra* L.), Mexican pinyon (*Pinus cembroides* Zucc. var. bicolor Little), Coulter pine (*Pinus coulteri* D. Don), Johann’s pine (*Pinus johannis* M.F. Robert), Italian stone pine (*Pinus pinea* L.) and Torrey pine (*Pinus torreyana* Parry ex Carrière) were sourced from different regions of New Zealand. The Torrey pine was grown in the Nelson region, the Armand and Coulter pines were grown in Canterbury, the Swiss stone and Johann’s pines were grown in Central North Island, the Mexican pinyon was grown in the Poverty Bay region and the Italian stone pine was grown in the Marlborough region of New Zealand. All of the regions where the nuts are grown, are on good quality agricultural land, previously either used for cropping, *i.e*., lucerne, oats or mixed pasture grass for livestock grazing [5]. The Italian stone pine is grown in sufficient quantity to supply a commercial market. The Marlborough region has a 29 year mean daily minimum/maximum air temperature of 5.9–17.4 °C and annual sunshine hours of 2420 [6]. At cone maturity the cones were dried and seeds subsequently sampled. One sample of pine nuts was purchased from the bulk bins in a local supermarket in Christchurch, New Zealand, there was no cultivar identification or country of origin, it was presumed to be imported.

### 2.2. Mineral Analysis

#### 2.2.1. Digestion Method

Duplicate samples of seeds were initially ground in a coffee mill (Sunbeam, model EM0400, China), then 0.3 g accurately weighed into a 75 mL Teflon PFA Kevlar-shielded digestion vessel (CEM Corporation, Matthews, NC, USA). To this 5 mL of a 4:1 nitric acid (69%): hydrogen peroxide (Aristar grade, Merck Ltd., KGaA, Darmstadt, Germany) acid mixture was added. The digestion vessels were then capped and the ground nuts were digested using a MARSXpress™ microwave digester (CEM Corporation, Matthews, NC, USA), programmed to ramp from ambient to 90 °C over 10 min then from 90 °C to 170 °C over 10 min where it was held for 10 min. Once cooled, 10 mL of deionized water (≥18 megohm) was added to make a final volume of 15 mL.

#### 2.2.2. Mineral Analysis

Mineral analysis was carried out on a Varian Axial 720 Inductively Coupled Plasma Optical Emission Spectrophotometer (ICP-OES, Varian, Palo Alto, CA, USA) with SP3 auto-sampler. Minerals were identified and quantitated using an ICP multi-element standard solution (CertiPUR, Merck, KGaA, Darmstadt, Germany) containing 23 elements or a single element standard, as required. Data and standard curves were processed using ICP-Expert™ II (Varian, Palo Alto, CA, USA). A water test standard and the ICP multi-element standard solution containing 0.50 μg/g of each element were run in triplicate with each batch to determine the standard error. The overall standard error was ±0.013 mg/kg. The limit of quantitation (LOQ) was performed using 10 times the standard deviation of the blank (5% nitric acid), for each mineral. Individual mineral LOQ values ranged from 0.12 to 12.24 μg/L, with a mean of 1.81 μg/L.

## 3. Results and Discussion

### 3.1. Mineral Profile

The mineral profile of the seven different pine nuts grown in New Zealand is shown in Table 1. Fourteen different minerals (Al, B, Ca, Cr, Cu, Fe, K, Mg, Mn, Na, Ni, P, S and Zn) were detected in all seven varieties. No detectable Al or Na could be found in the sample of *Pinus coulteri* D. Don. and no Cr was detected in the samples of *Pinus armandii* Franch and *Pinus cembra* L., analysed in this study. In comparison to the currently presumed imported pine nut purchased from the local supermarket, of an unknown variety, the New Zealand grown pine nuts had higher mean values for Ca, Cu, Fe, K, Mg, Na, P, S, and Zn. The mean New Zealand value for Al was lower. Higher mean New Zealand values for B, Cr, Ni and a lower mean value for Mn, compared to the supermarket nut are to be noted.

**Table 1:** Mineral content (mg/kg) of seven different pine nuts grown in New Zealand (*n* = 2).

Common name (scientific name)	Al	B	Ca	Cr	Cu	Fe	K	Mg	Mn	Na	Ni	P	S	Zn
Armand pine	33.2	41.9	322.3	nd	30.7	150.5	13,590.2	6978.4	127.4	6.4	4.1	16,790.9	5826.7	200.5
(*Pinus armandii* Franch)														
Swiss stone pine	32.9	27.8	172.8	nd	15.3	89.6	11,478.9	4883.8	55.4	2.5	1.8	11,216.5	5019.5	94.2
(*Pinus cembra* L.)														
Mexican pinyon	29.8	65.1	156.1	0.2	17.3	72.1	7857.7	3958.1	75.8	1.2	5.9	8621.2	2496.7	71.2
(*Pinus cembroides* Zucc. var. bicolor Little)														
Coulter pine	nd	14.5	273.2	0.3	28.3	117.0	14,531.5	7500.2	88.9	nd	2.7	19,268.9	7384.7	266.5
(*Pinus coulteri* D. Don)														
Johann’s pine	38.5	78.3	125.7	0.3	13.5	71.5	8860.9	3456.4	49.9	5.4	3.5	7892.6	2482.6	62.9
															(*Pinus johannis* M.F. Robert)
Italian stone pine	32.1	39.2	486.2	0.4	20.2	142.8	14,538.7	7880.7	116.9	3.4	10.4	19,817.8	7891.8	166.4
(*Pinus pinea* L.)														
Torrey pine	18.8	30.2	264.7	0.6	35.9	81.9	12,597.0	7493.0	147.6	3.2	12.8	18,208.3	4976.5	184.6
(*Pinus torreyana* Parry ex Carrière)														
Mean	30.9	40.4	258.7	0.4	23.0	103.6	11,922.1	6021.5	94.6	3.7	5.9	14,545.1	5154.1	149.5
Supermarket pine nuts	53.3	22.8	180.7	nd	21.1	62.6	8052.6	3521.4	134.7	nd	3.8	7556.2	2807.5	85.6

nd = none detected.

The most abundant mineral for five of the pine nuts was phosphorus, and for *Pinus cembra* L. and *Pinus johannis* M.F. Robert, potassium was the most abundant, but not by a large amount. This is consistent with previous investigations, which identified P as the most abundant mineral in Portuguese grown pine nuts [2] and K in Turkish samples [4]. In both cases P and K predominate, as with the New Zealand grown pine nuts. The differences in the minerals are probably due to soil conditions, climate and growing practices.

The mineral profile results for the New Zealand and Portuguese [2] *P. pinea* (Table 2) are more comprehensive as both were performed using ICP-OES. This method enables the collection of data on as many minerals as the equipment is calibrated for and results from a single injection into the ICP-OES. The mineral results from other research [4,7,8] have used older but still reliable methods for mineral analysis.

**Table 2:** Comparison of New Zealand grown Italian stone pine (*Pinus pinea*) mineral content (mg/kg) with published data from six different countries.

	Al	B	Ca	Cr	Cu	Fe	K	Mg	Mn	Na	Ni	P	S	Zn	Reference
New Zealand	32.1	39.2	486.2	0.4	20.2	142.8	14,538.7	7880.7	116.9	3.4	10.4	19,817.8	7891.8	166.4	(this study)
Portugal	-	24.4	319	-	34.3	111.2	8920	5332	160.5	10.1	-	11,300	4854	111.2	[2]
Spain	5.1	-	-	0.3	20.4	22.3	-	-	-	-	0.11	-	-	50.2	[7]
Turkey	-	-	138	-	15	102	7,130	3250	69	117	-	5120	-	64	[4]
Crete	-	-	140	-	-	44	-	-	-	-	-	5150	-	-	[8]
USA	-	-	160	-	13.2	55.3	5970	2510	88.0	20	-	5750	-	64.5	[9]
Australia	-	-	110	-	12	41	6000	2300	69	30	-	5600	1100	53	[10]

- = not reported.

The data referred to from the USA and Australia [9,10] (Table 2) are from nationally collected databases, where the data is often an overall mean from several sources and the provenance of the data is limited.

Since stone pine (*P. pinea*) had the best mineral profile of all the *Pinus* varieties grown in New Zealand its mineral content is compared to published data from six different countries, using the same cultivar where possible (Table 2), some of the data from USA and Australia [9,10], is not solely from *P. pinea*. In all cases, except for Cu, Mn and Na, the mineral content was higher. This suggests that the environment in Marlborough is conducive to the growth of quality products. The Marlborough region annual sunshine hours are 2420 [6], which is the highest value observed in New Zealand and is comparable to annual sunshine hours in regions of the Mediterranean. The soils in the Marlborough region are a mixture of older loess and post-glacial sheet outwash soils, ranging from 100 to 300 metres above sea level [11]. Features of loess soils are fertility, low organic matter and high cation exchange capacity, which enables efficient absorption of nutrients by the trees.

Table 3 compares the results obtained for the mineral content of New Zealand stone pine with published data obtained for some common tree-nuts. In general the mineral contents are largely comparable to the values observed in the common tree-nuts. However for the elements, Al, Fe, K, Mg, Mn, Ni, P and Zn, New Zealand stone pine appears to be a better source of these elements. There are no published values of S in tree-nuts to compare our results to. The very high Na levels observed in the data for cashew nuts suggest that salt was added during processing.

**Table 3:** Comparison of New Zealand Italian stone pine (*Pinus pinea*) mineral content (mg/kg) with published data for some common edible tree-nuts.

Common name	Al	B	Ca	Cr	Cu	Fe	K	Mg	Mn	Na	Ni	P	S	Zn
Stone pine	32.1	39.2	486.2	0.4	20.2	142.8	14,538.7	7880.7	116.9	3.4	10.4	19,817.8	7891.8	166.4
Almonds [12]	-	-	5392.4	0.94	23.7	71.5	-	5424.1	25.8	-	-	-	-	49.7
Brazil [12]	-	-	7324.8	1.34	59.4	74.3	-	9678.5	3.4	-	-	-	-	110.3
Cashew [13]	-	-	173.0	-	10.7	153.6	2967.7	2725.5	50.7	4557	-	287.1	-	38.6
Hazelnut [14]	-	45.0	806.3	0.9	11.9	20.1	8105.5	655.5	33.2	264.1	0.8	3203.3	-	8.9
Macadamia [12]	-	-	3376.1	1.3	18.9	68.1	-	4886.5	88.6	-	-	-	-	38.4
Pecan [12]	-	-	2088.4	2.0	35.5	105.8	-	4197.0	196.6	-	-	-	-	137.8
Walnut [15]	2.3	-	619.2	5.3	22.4	46.0	4140	2,166	115.4	7.4	-	3460	-	26.2

- = no data reported.

### 3.2. Nutritional Implications

A feature of the seven *Pinus* nuts evaluated was the low Ca:P, Ca:Mg and Na:K ratios, with respective means of 0.017, 0.041 and 0.0003. However, these ratios are common to most edible tree nuts, such as walnuts, hazelnuts and pecans.

Table 4 is based on the Qualified Health Claims (QHC) of the FDA [19] which “suggests but does not prove 1.5 ounces (42 g) per day of most nuts” as being a suggested amount to achieve reduction in heart disease. The consumption of 50 g of pine nuts per day can meet or exceed the RDI of Cu, Mg, Mn, P and Zn (Table 4). This amount of pine nuts can also provide a reasonable amount (39%–89%) of Fe. The contribution of Al and B to the RDI is low (1.9%, 9.8% to 17%, respectively).

**Table 4:** Recommend daily intake (RDI) of minerals and percentage provided by the consumption of 50 g of New Zealand grown Italian stone pine (*Pinus pinea*) nuts.

Mineral	RDI ^1^	% of RDI
	(mg/day)	(50 g nuts/day)
Al	84 ^2^	1.9
B	11–20 ^3^	9.8–17
Ca	1000–1300	1.8–2.4
Cr	25–35 ^4,5^	100–140
Cu	1.2–1.7 ^4^	130–184
Fe	8–18	39–89
K	2800–3800 ^4^	19–26
Mg	310–420	94–127
Mn	5.0–5.5 ^4^	107–117
Na	460–920 ^4^	0.02–0.05
P	1000	96
Zn	8–14	59–104

^1^ Recommended daily intake, based on New Zealand adult male and female aged from 19 to 70 years [16]; ^2^ Tolerable weekly intake (TWI), for 70 Kg adult person [17]; ^3^ Tolerable upper limit (UL) [18]; ^4^ Adequate intake (AI) [16]; ^5^ μg/day.

## 4. Conclusions

The New Zealand grown pine nuts analysed in this study are a good source of dietary minerals, particularly Cu, Fe, K, Mg, Mn, P and Zn when compared to other commonly eaten tree-nuts. In comparison to pine nuts grown in other countries New Zealand grown pine nuts are a richer source of many minerals and provide a good source of Cu, Fe, Mg, Mn, P and Zn. The positive mineral profile of pine nuts grown in New Zealand may be due to good growing conditions for the trees. It would be beneficial to recommend more widespread consumption of New Zealand pine nuts.
